# A Review on the Current State of Microcapsule-Based Self-Healing Dental Composites

**DOI:** 10.3390/jfb15060165

**Published:** 2024-06-16

**Authors:** Xiaoxi Wang, Tian Ding

**Affiliations:** School of Stomatology, Cheeloo College of Medicine, Shandong University & Shandong Key Laboratory of Oral Tissue Regeneration, No. 44-1 Wenhua Road West, Jinan 250012, China; doctorwxx16@163.com

**Keywords:** microcapsule, microcrack, self-healing, dental resin composite, antibacterial property, mechanical property

## Abstract

Resin-based dental composites, commonly used in dentistry, offer several advantages including minimally invasive application, esthetically pleasing appearance, and good physical and mechanical properties. However, these dental composites can be susceptible to microcracks due to various factors in the complex oral environment. These microcracks can potentially lead to clinical restoration failure. Conventional materials and methods are inadequate for detecting and repairing these microcracks in situ. Consequently, incorporating self-healing properties into dental composites has become a necessity. Recent years have witnessed rapid advancements in self-healing polymer materials, drawing inspiration from biological bionics. Microcapsule-based self-healing dental composites (SHDCs) represent some of the most prevalent types of self-healing materials utilized in this domain. In this article, we undertake a comprehensive review of the most recent literature, highlighting key insights and findings related to microcapsule-based SHDCs. Our discussion centers particularly on the preparation techniques, application methods, and the promising future of self-healing microcapsules in the field of dentistry.

## 1. Introduction

Restorative materials play a crucial role in the treatment of dental caries. Dental amalgam was once a popular material for dental restorations because it was affordable and had many useful properties, such as excellent durability and strength. Despite the benefits of amalgam fillings, their safety has been a highly controversial topic for many years [1,2,3]. In the 1960s, bisphenol A–glycidyl methacrylate (Bis-GMA) was synthesized for the first time, which initiated the development of modern polymeric composite [4]. It also provided a better choice of restorative materials. Additionally, the use of amalgam in dentistry has been phased down since then [5]. In addition, a globally legally binding treaty known as the Minamata Convention on Mercury has accelerated the phasing out of dental amalgam [6]. Nowadays, resin-based composites are widely used in oral clinical work due to their excellent esthetics and easy operation [7,8]. However, in complicated oral conditions, the macromolecular chains in the composite resin matrix can undergo homolysis or heterolysis under various physicochemical and microbiological factors, resulting in imperceptible microcracks [9]. Microcracks gradually expand and integrate, which can ultimately cause the material to fracture and affect the effect of clinical therapy [10,11]. Therefore, it is necessary to explore effective methods to solve the above problems, and self-healing polymer materials based on biological bionics emerge as the times require. In the past two decades, self-healing polymers have been extensively reported in engineering, aerospace and other fields [12,13,14,15], which has also inspired attempts in the field of dentistry.

**Figure 1:**
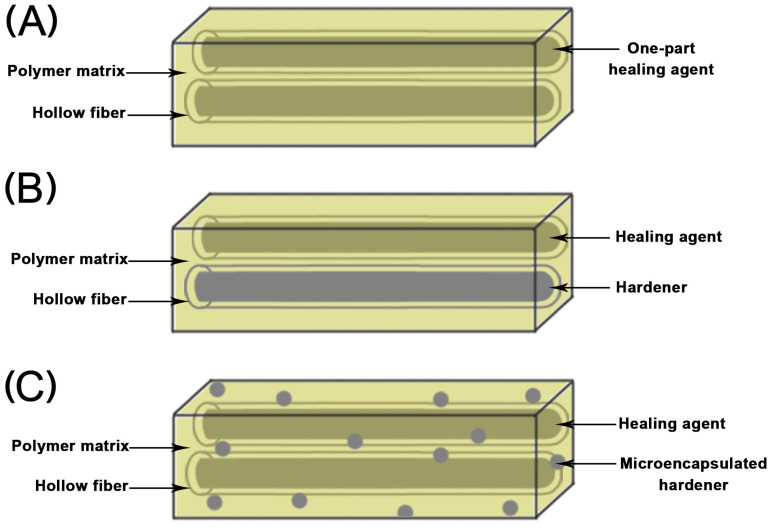
Diagram of self-healing materials containing hollow fibers. (**A**) One-part healing agent being encapsulated within the hollow fiber. (**B**) Two-part resin with the healing agent and hardener being encapsulated separately in the hollow fiber. (**C**) Healing agent being encapsulated within the hollow fiber and catalyst being distributed evenly within the polymer matrix.

Self-healing polymers are a class of intelligent materials that can self-sense damage and self-repair, simulating the self-cure phenomenon of organisms [16]. When microcracks occur, healing agents can seal and stop crack propagation by exposing themselves to the catalyst or changing inherent chemical bonds, thus maintaining the integrity of the matrix structure. Generally, self-healing materials can be divided into intrinsic and external categories based on their underlying mechanism. Intrinsic self-healing materials work based on their own chemical structural characteristics. They can achieve their self-healing function through chemical interactions between reversible covalent and non-covalent bonds [17]. However, microcracks commonly occurs under certain external conditions, so there are some obstacles to these polymers’ application in dentistry [18,19]. External self-healing materials primarily rely on a matrix-embedding vascular network or microcapsules containing healing agents to achieve self-healing ability. Vascular network self-healing materials utilize pre-embedded hollow fibers to transfer self-healing agents, generating bonding and repair cracks, which simulate the vascular structure in biology, to achieve self-healing capability [20]. The diameter of hollow fibers is minute, usually in the range of 40 to 200 μm. The arrangement of fibers is varied, and can be vertically crossed, parallel, or inclined. Vascular network self-healing materials can typically be further divided into various categories based on the composition of the healing agent inside the hollow fiber [21] (Figure 1).

**Figure 2:**
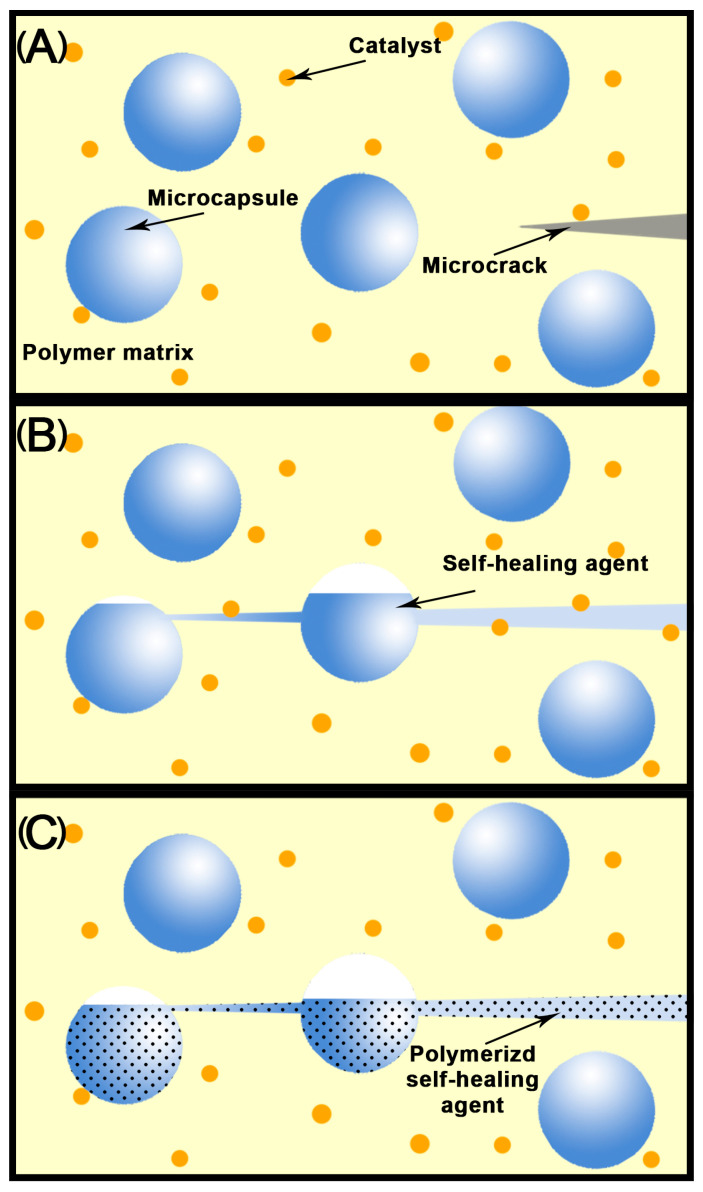
Principal diagram of microcapsule-based self-healing materials. (**A**) Perception stage. (**B**) Delivery stage. (**C**) Reaction stage.

Microcapsule-based self-healing materials function in the following ways [22] (Figure 2). Firstly, microcracks start to form and expand into the microcapsules. Then, the delivery stage begins where the expanding cracks puncture the microcapsules and the agents are delivered to the crack surface through a siphon effect. Ultimately, the reaction phase takes place where the healing agents react and polymerize with pre-embedded catalysts in the matrix to complete self-recovery from damage.

Among the above self-healing materials, microcapsule-based self-healing materials have been considered to have the greatest potential, because of the unchanged characteristics of the polymers, the mature synthesis process, and their easy implantation [22]. Thus, extensive research and reports relevant to microcapsule-based self-healing dental composites (SHDCs) have been conducted [23]. The purpose of this review is to gather information from and reflect upon the cutting-edge research on microcapsule-based SHDCs, particularly focusing on the relative points of microencapsulation, as well as the applications and prospects of self-healing microcapsules in dentistry.

## 2. Microencapsulation Technology

Microencapsulation is a technology of encapsulating active liquids, solids, or gases in micron- or nano-sized spherical products that are designed to gradually release their contents under specific conditions [24]. Thus, the following requirements are essential in order to enable self-healing materials to work with high performance: (1) Shell materials should exhibit suitable thickness and intensity to ensure that microcapsules can withstand pressure during processing and rupture timely after sensing cracks. (2) Agents should have excellent fluidity and reaction rate to guarantee that the liquid can flow to the break accurately. (3) The size of microcapsules should not be too large or too tiny; otherwise, it would impair the self-healing efficiency. (4) Stable chemical and physical properties are required so that the microcapsules can be stored for a long time. (5) Mechanical properties of microcapsules are supposed to match those of the matrix, exhibiting positive material compatibility. (6) The preparation methods are expected to be manipulated easily and come with high encapsulation efficiency, allowing for mass production. To meet the aforementioned requirements and obtain the desired microcapsules, suitable core and shell materials, effective preparation methods and appropriate characterization approaches are used in complement to one another and not a single one can be omitted.

### 2.1. Selection of Core and Shell Materials of Microcapsules

The typical structure of microcapsules is a core–shell design, consisting of a core material (self-healing agent) and a shell material (polymeric shell) surrounding it. In 2001, White et al. first presented a capsule-based self-healing system in which the self-healing agent dicyclopentadiene (DCPD) was successfully encapsulated in the shells of urea–formaldehyde (UF) [25]. Grubbs’ catalyst was first positioned in an epoxy resin matrix. Once microcracks were created, the microcapsules burst and released DCPD, which came in contact with the catalysts, triggering a ring-opening metathesis reaction. Then, polymerization products were polymerized with the epoxy resin matrix to repair cracks, and ultimately achieved self-healing properties. Since then, the microcapsule-based self-healing system containing PUF-DCPD microcapsules and Grubbs’ catalysts has been extensively used and continuously refined [16]. Currently, one of the most commonly used wall materials is amino resin [26], including poly(urea-formaldehyde) (PUF) [27,28], poly(melamine-formaldehyde) (PMF) [29], poly(methyl-methacrylate) (PMMA) [30,31,32] and polyurethane (PU) [33]. In addition to classic DCPD and alicyclic derivatives, other materials such as polymerizable agents, organic solvents, dry oils and epoxy resins are available for the core [34].

### 2.2. Preparation Methods of Microcapsules

**Figure 3:**
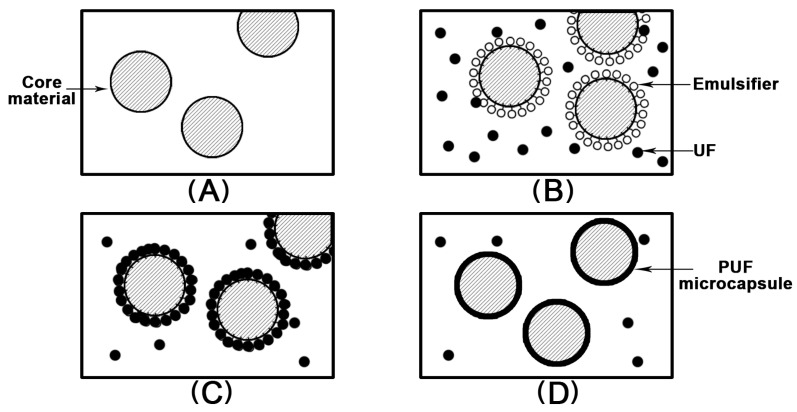
Preparation of PUF microcapsules via in situ polymerization. (**A**) Core material being dispersed in solution. (**B**) Emulsification and precipitation of UF from solution. (**C**) Gradual deposition of UF on core droplet surface. (**D**) Formation of capsule wall and generation of PUF microcapsules.

Based on the formation mechanism of the shell, the fabrication process of microcapsules can be roughly divided into three types: the physical method, chemical method and physicochemical method [35]. For physical treatment, spray drying [36], solvent evaporation [37] and air suspension [38] are commonly used. The chemical method contains include in situ polymerization [25,39,40], interfacial polymerization [41,42] and piercing methods [43]. Physicochemical methods, also known as phase separation methods, consist of oil phase separation [44], simple coacervation [45] and complex coacervation methods [46]. Among the above methods, in situ polymerization is the most effective, easy-to-control and easy-to-perform method for the preparation of self-healing microcapsules [47]. It can allow us acquire desired microcapsules by adjusting the stirring rate without complicated equipment [47]. The in situ polymerization method comprises two main steps (Figure 3). The first stage is emulsification of the core material (the healing oil). With the participation of the emulsifier, the healing oil in the core material is dispersed into tiny droplets under mechanical stirring or ultrasonic action to form an oil-in-water emulsion. The type and amount of emulsifier, as well as the time and speed of emulsification, have a close relation to the surface morphology and particle size of the microcapsules. Encapsulation of the wall material is the next stage. The monomers or their prepolymers polymerize with the catalysts on the droplet surface to generate polymers. Then, the microcapsules are produced when the substance is deposited on the surface of core materials. In this process, factors that can affect the encapsulation efficiency and the performance of microcapsules such as the type of wall material, core–wall ratio, and stirring rate must be taken into account [26].

The preparation process of DCPD microcapsules via in situ polymerization was thoroughly studied by Brown et al. [48]. The microcapsules with particle sizes of 10–1000 μm were prepared in the stirring rate range of 200–2000 r/min, and the average particle size was inversely proportional to the stirring rate. The thickness of the shell ranged from 160 nm to 220 nm, providing excellent storage and release properties for the application of self-healing materials. During the preparation of microcapsules, UF particles were formed and deposited on the surface of the microcapsules, increasing the shell roughness of the microcapsules, which enhanced the mechanical adhesion between capsules and the matrix. Since PUF was proved to be deficient in heat resistance, sealing and mechanical strength, Yuan et al. selected PMF as the shell material and managed to encapsulate highly active pentaerythritol tetrakis-3-mercaptopropionate (PETMP) via in situ polymerization [40]. The optimum synthesis conditions were determined to be a reaction temperature of approximately 50 °C, a pH within the range of 2.9–3.2, and a core/shell feed weight ratio of around 2.3. Not only was the core material content sufficient, but the shell thickness and strength reached a balance under these conditions. Fracture strength and fatigue tests indicated that the core retained high reactivity even after incorporating the microcapsules into the resin matrix composites and being placed at 250 °C for 24 h.

### 2.3. Characterization of Microcapsules

The analysis of microcapsules is a part of the microencapsulation process. It is vital to characterize the prepared microcapsules prior to their formal usage and provide guidance for the subsequent application of self-healing materials through physical, chemical, structural and biological properties.

#### 2.3.1. Surface Topography

The size of microcapsules is too small at the micron scale or even nanoscale to see with the naked eye. Therefore, an optical microscope (OM) and a scanning electron microscope (SEM) are required for observation. The OM can allow us to observe the formation of microcapsules, but only in a 2D image. An SEM is used to evaluate the surface morphology of microcapsules in a 3D image. It judges the appropriateness of the process conditions based on the smoothness of the shell, as well as whether there is adhesion, collapse or fold.

#### 2.3.2. Physical Characteristics

##### Particle Size and Distribution

There is no denying that the size of microcapsules has a significant impact on the efficiency of self-healing. The most common detection method is to use an OM with a measuring system or study platinum-treated samples under SEM photogrammetry to obtain the particle size and distribution range by averaging the diameters of hundreds of microcapsules. However, this method is quite cumbersome and hard to apply. Nowadays, a more accurate method is to utilize a laser particle size analyzer, which is based on the principle of indirectly investigating the average particle size and distribution via the measurement of scattering light energy [49].

##### Encapsulation Efficiency

Encapsulation efficiency is an important parameter indicating the effectiveness of microencapsulation. The gravimetric method is the most used method, and involves weighing the mass of microcapsules before and after drying, and then using an empirical formula to calculate the results [50]. Thermogravimetric analysis (TGA) testing can be performed on microcapsules as well [28]. The specific steps involve performing TGA on the wall material, core materials and microcapsules. Then, the following steps include comparing the decomposition weight loss temperatures and weight loss masses on the TGA curve to preliminarily determine the microencapsulated ratio of the core materials [28].

##### Mechanical Property

The mechanical properties of microcapsules primarily depend on the strength of the shell, which determines whether microcapsules have the ability to withstand pressure without breaking during processing. The single-microcapsule compression test has become the most widely used measure. The fundamental concept of this test is to sandwich a single microcapsule between two parallel surfaces comprising a microscope sample stage and a force sensor probe. This setup is designed to induce a specific level of deformation or breakage in the microcapsule at a predetermined rate, while monitoring the amount of force applied to the microcapsule [51,52,53].

#### 2.3.3. Chemical Characterization

##### Fourier Transform Infrared (FTIR) Spectroscopy Analysis

Different types of chemical bonds and molecular architectures can lead to different vibration modes and frequencies. FTIR is employed to characterize the wall material, core material and microcapsules separately, and compares the infrared spectrum of the three. Changes in functional groups, encapsulation efficiency and polymerization extent are evaluated based on the relative intensity of absorption peaks of different characteristic groups [54].

##### X-ray Diffraction (XRD) Analysis

When X-rays irradiate on a crystal, X-ray diffraction occurs due to the structure arrangement of crystals. The direction and intensity of the diffracted light are related to the crystal lattice structure. This method qualitatively analyzes the phase composition of the sample by comparing the X-ray diffraction spectra between the test sample and reference material [55].

##### Thermal Stability

Thermal stability refers to the deformability of the material under temperature influence. The smaller the deformation, the higher the thermal stability. There are two methods commonly applied in material thermal analysis: differential scanning calorimetry (DSC) and TGA. DSC measures the energy or power difference between the material and reference as a function of temperature or time when under a procedure with temperature control [56]. On the other hand, TGA is performed to examine the relationship between the specimen weight and temperature or time under programmed temperature control.

#### 2.3.4. Cytotoxicity

The requirements for microcapsules in biological and clinical applications differ from those in the chemical, industry and building fields. Thus, it is crucial to consider biosafety issues, which demand that microcapsules have no cytotoxicity and no negative influences on the human body. The MTT assay is a method for detecting the survival and growth of cells, and has become the most commonly used method. It is mainly used for testing the cytotoxicity of drugs or other intervention measures on cells cultured in vitro [57].

## 3. Applications of Self-Healing Microcapsules in Dental Composites

In order to overcome the drawbacks of conventional dental resin-based composites, such as matrix fracture and secondary caries in practical clinical applications, scholars in the dental field have introduced the self-healing system based on the microcapsule model into dental polymers. This has promoted the development of SHDCs, followed by many promising achievements in various research studies [23].

### 3.1. Development of Dental Self-Healing System Based on Microcapsules

In 2010, Wertzberger et al. consulted White’s self-healing model [25] for the first time by incorporating PUF-DCPD microcapsules and Grubbs’ catalyst into a dental composite resin containing a high level of inorganic fillers (55 wt%), and obtained 57% self-healing efficiency [58]. These results showed that despite the high filling nature of dental composites, mechanical properties could be restored via the crosslinking and curing reactions of implanted monomers and catalysts. Next, melamine was added to the shell for modification, expecting this to confer a stronger mechanical combination, in Then et al.’s survey [59]. This finding proved that microcapsules could be evenly dispersed in the resin matrix, increasing the possibility of microcracks extending into microcapsules. Meanwhile, mechanical performance testing confirmed that adding low-content microcapsules has no impact on mechanical properties. This discovery provided a new modification method for the application of UF microcapsules in dental polymer materials.

DCPD had the advantage of being affordable and easy to encapsulate, but there were issues with biosafety [60]. Simultaneously, Grubbs’ catalysts were costly and toxic [61], which limited the application of DCPD–Grubbs’ systems in the dental field. As a result, researchers have begun to better explore suitable self-healing systems with microcapsules for dental clinical applications. Ouyang et al. prepared PU-TEGDMA nanocapsules using miniemulsion polymerization and added the microcapsules into commercial dental resin adhesives [57]. The research results indicated that these microcapsules had good drug loading properties and biocompatibility. Furthermore, the resin adhesive containing nanocapsules exhibited stronger adhesive properties compared with the control group. However, to achieve self-healing function in dental materials, further investigation was needed on the content of self-healing agents within the microcapsules and the instant rupture of the microcapsules after cracking. Based on this point, Wu et al. synthesized a novel self-healing microcapsule using TEGDMA-DHEPT as the core material and PUF as the shell material [62]. It was demonstrated that the microcapsules were effectively encapsulated and fairly heat-stable. Subsequently, different contents of microcapsules were incorporated into resin composites, and the results confirmed that the addition of 15 wt% microcapsules could achieve 65% self-healing efficiency without damaging the original mechanical strength, which was generally consistent with Wertzberger et al.’s research [58]. Ning et al. prepared PUF microcapsules measuring three sizes: large, medium and small [63]. Then, different sizes of microcapsules and different concentrations of initiators were inserted into commercially flowable resin matrix composites. It turned out that the fracture toughness showed an increased tendency with the increase in microcapsule sizes and initiator concentrations. Adding larger microcapsules (198 ± 43 μm) allowed 76% self-healing efficiency to be reached, which might have been related to the easier fracture, higher fluid pressure and greater mechanical interlocking of the larger microcapsules [27]. However, the long-term performance of SHDCs with large microcapsules remained to be investigated.

Moreover, achieving self-repairing functionality while maintaining the original mechanical properties of dental resins was also a major concern. Researchers have attempted to enhance dental resins by adding various materials, including nano- and micro-sized fillers, whiskers, and fibers. Incorporating silane-treated nanohydroxyapatite-filled zinc oxide could significantly improve the physical, mechanical, and thermal properties of dental composite materials [64]. It was pointed out that addition of an appropriate amount of silica filler in composites can enhance mechanical properties [65,66]. Althaqafi et al. investigated the effect of adding TEGDMA self-healing microcapsules with varying contents (0–10 wt%) and 20 wt% silica nanoparticles on the properties of dental composites [67]. The results exhibited that the transmittance, conversion degree, hardness and elastic modulus of the SHDC were almost unchanged, while the bending strength decreased. It was suggested that mechanical performance-enhancing self-healing composites were able to be synthesized by exploring more appropriate filler contents.

Researchers have continuously innovated and improved self-healing systems to synthesize microcapsules that are closer to having actual clinical applications, as well as further improved the mechanical properties of SHDCs. Because of the high polymerization shrinkage of TEGDMA, Moreira et al. attempted to develop a new self-healing system [68]. In this study, three types of self-healing microcapsules involving various agents and polymerization regulators were prepared: TC_DHEPT_ (TEGDMA, DHEPT), BTC_DHEPT_ (Bis-GMA, TEGDMA, DHEPT), and BTC_BPO_ (Bis-GMA, TEGDMA, BPO). Based on previous research [69,70], a ratio of 7.5 wt% microcapsules was introduced into resin composites. It has been confirmed that the group containing BTC_DHEPT_ and BTC_BPO_ showed the ability to promote crack healing [68], but this was much weaker than that in the TC_DHEPT_ group. Thus, the proportion and long-term performance of the Bis-GMA-TEGDMA self-healing system required further research. An article pointed out that the addition of N,N-dimethylacrylamide (DMAM) (a high-toughness component) into high-crosslinked dental polymers could improve their mechanical properties and capacity to absorb continuous dynamic stress [71]. Fugolin et al. introduced DMAM, as one of the self-healing agents, into the TEGDMA self-healing system [72]. To explore the changes in the fatigue–crack propagation behavior of such system, a more clinically relevant test method known as the double-torsional fracture toughness technique was operated. This method was able to provide more information on crack initiation and extension [73]. Furthermore, the results indicated that the self-healing system incorporating DMAM exhibited 77–94% toughness recovery and reversible bonding ability [72], which made it possible for DMAM to replace TEGDMA partially. Furthermore, research by Ahangaran et al. found that PMMA had good development potential as the shell material in a self-healing system [74,75] and thus introduced PMMA into dental composites [76]. Therefore, a novel self-healing microcapsule with PMMA as the shell and TEGDMA as the healing agent was synthesized by means of the solvent evaporation method. Adding microcapsules into a mixture of acrylic resin and nano-SiO_2_ modified with 3-methacryloxypropyltrimethoxysilane (MPS), it was proven that 78–121% self-healing efficiency was achieved without a significant decrease in mechanical properties and also without cytotoxicity [76]. SiO_2_ was also selected as the microcapsule shell for the applications of SHDCs. An SHDC was designed by using aqueous polyacrylic acid, the main component of the glass ionomer cement (GIC) liquid phase, as a healing agent according to the reaction principle of GIC [77]. The main component of GIC powder, strontium fluorosilicate particles, was placed in the resin matrix. When cracks appeared, the microcapsules ruptured, causing acid–base reactions between the liquid and powder, via which the crack was repaired, realizing a self-healing process. The average self-healing efficiency was up to 25%, when the content of microcapsules was between 5 and 10 wt% [77]. This provided evidence that this new self-healing system could be introduced into dental composites.

Additionally, previous studies proved that microcapsule surfaces dealing with a silane coupling agent could improve the joining strength between the capsule and the resin matrix [78,79], which was one of the key factors that reinforced the self-healing effect. According to the research, Yahyazadehfar et al. prepared two sets of GIC microcapsules that were treated with different silane coupling agents [80]. One set was prepared using methacrylate silane (MA-silane) and the other was prepared with hydroxyl silane (OH-silane). The results showed that the incorporation of up to 5 wt% MA–silane microcapsules by mass performed the best in terms of fracture toughness and self-healing efficiency, and that the rupture speed was able to reach five times that of the untreated microcapsules [80]. This indicated that silanization played an active role in SHDCs’ acquisition of clinically applicable mechanical properties and excellent self-healing properties at the same time. Sharma et al. developed silanized microcapsules, with an emphasis on the influence of SHDCs in terms of mechanical properties and dynamic mechanical behavior after adding microcapsules with different weight percentages [81]. The research demonstrated that the addition of silanized microcapsules led to an increase in the hardness and flexural strength of the composites. Rozza et al. used MPS to perform surface silanization of PUF microcapsules and investigated the effect of incorporating silanized and non-silanized microcapsules with different mass fractions into dental composites [82]. These results proved that the group containing silanized microcapsules did not compromise flexural strength and achieved higher self-healing efficiency than the non-silanized group. This phenomenon might have been due to the better interfacial bonding between the microcapsules and the resin matrix [78]. The potential of silanization treatments in enhancing the self-healing capacity of dental composites was also further confirmed. Table 1 provides a detailed summary of dental self-healing systems based on microcapsules.

### 3.2. SHDCs with Multiple Functions

The main causes of clinical restoration failure are secondary caries and restoration fractures [83]. Among them, dental caries are strongly associated with the attachment of plaque. Therefore, satisfactory dental composites should not only repair cracks but also reduce oral biofilm attachment. It has been proven that dental materials containing 2-methacryloyloxyethyl phosphorylcholine (MPC) are able to reduce the adsorption of proteins [7,84]. Chen et al. incorporated MPC into dental composite resins composed of PUF microcapsules and successfully developed a formulation design with dual functions of self-healing and protein resistance [85]. This finding created new ideas for research on anti-caries dental restorative materials. Ahangaran et al. prepared self-healing microcapsules by using PMMA as the shell and added them to acrylic resin, achieving satisfactory self-healing efficiency [76]. Moreover, the smooth and non-porous surface of the PMMA microcapsules made it difficult for *Streptococcus mutans* to attach, colonize and spread on the surface of the acrylic-based self-healing material. Therefore, the SHDCs containing PMMA microcapsules also showed positive antibacterial properties [76].

Wu et al. mixed quaternary ammonium salt monomer–dimethylaminododecyl methacrylate (DMAHDM), amorphous calcium phosphate (NACP) nanoparticles and PUF microcapsules into dental composites [69]. The experimental outcome indicated that the mechanical strength of the composite resin was hardly affected when incorporating 7.5 wt% microcapsules. At the same time, 65–81% self-healing efficiency and excellent antibacterial effects were achieved, realizing the triple functions of antibacterial, remineralization and self-healing properties in the SHDC [69]. In addition, there was a study on the addition of DMAHDM, NACP and microcapsules to dental adhesive resins [86]. Via experimental confirmation, a new antimicrobial resin adhesive with self-healing functions that could be used for clinical applications was developed [64,87,88,89].

Furthermore, it can not be ignored that the existing self-healing materials had the disadvantage of fragile and single-functionality shells. Yao et al. synthesized self-healing microcapsules modified with nano-antibacterial inorganic fillers, which showed improvements in surface roughness, modulus and hardness [90]. The incorporation of 7.5 wt% microcapsules in dental composites resulted in 69% self-healing efficiency and effective antimicrobial properties [90]. To further explore the performance of these novel microcapsules, Yao et al. conducted more research. It was confirmed that the SHDCs achieved excellent antibacterial and self-healing properties without affecting hydrophilicity, mechanical properties or cytotoxicity [91,92]. Unlike the case for the materials found in the existing literature, this novel SHDC can help the multifunctionality of materials be realized simply via the addition of nanoparticle-modified microcapsules, showing great potential in the development of multifunctional SHDCs. Then, to meet clinical needs, a novel type of dental resin was prepared, containing an anti-shrinkage mixture of an expanding monomer and an epoxy resin monomer (1:1, referred as UE). In addition, microcapsules with antibacterial fillers deposited on the surface were also included [93]. The experimental results indicated that the addition of 7.5 wt% microcapsules and 20% UE to the resin not only reduced the colony-forming units (CFU) by 3 orders of magnitude and obtained 71% self-healing efficiency, but also reduced polymerization volume shrinkage by 30.12% compared with the control [93]. This suggested that an SHDC with three functions, low-shrinkage, antibacterial, and self-healing properties, has been developed, presenting new possibilities for the clinical application of self-healing materials.

### 3.3. Durability and Fatigue Resistance of SHDCs

SHDCs were exposed to moist oral environments and were subjected to daily chewing forces from occlusion. The combination of high humidity, cyclic loading, and abrasion made the polymers highly susceptible to damage from microcracks, which greatly affected the materials’ service lives [94]. Consequently, this required self-healing composites to have sufficient durability and fatigue resistance to ensure their promotion and application in practical clinical work.

In order to study the aging of composite resins containing microcapsules, a specimen containing 7.5 wt% microcapsules was immersed in water for 6 months by Wu et al. [95]. After aging treatment, it was found that the composites almost had the same self-healing effect as before and achieved self-repairing in water, which was more conducive for oral applications. Then, the self-healing concept was also applied to dental resin cement in common use. Wu et al. synthesized self-healing dentin bonding cement combined with PUF-TEGDMA microcapsules [96]. This study confirmed that cement with 7.5 wt% microcapsules had excellent adhesion and self-healing efficiency. Even after 6 months of water immersion, it still exhibited excellent self-healing performance [96]. In addition, Ravandi et al. investigated the mechanical and water aging properties of self-healing nanocomposites containing nanoclay and PUF nanocapsules prepared at different stirring rates [97]. These results indicated that the optimal mechanical properties could be obtained when the stirring rate was 1300 rpm and the incorporation of microcapsules was 7.5 wt%. In these conditions, a self-healing efficiency of 54–58% was achieved. Testing the self-healing performance after 90 days, there was no significant decrease [97], which also confirmed that this novel self-healing dental nanocomposite had sufficient durability.

To investigate the fatigue resistance of the SHDC, Kafagy et al. developed four types of PUF microcapsules containing common dental healing agents using in situ polymerization [98]. The results showed that the fatigue resistance of the SHDC improved, but the self-healing efficiency only reached 40%, which might have been due to the insufficient release of a self-healing monomer or the incomplete resetting of the two fracture ends [99]. Moreover, Yahyazadehfar et al. functionalized microcapsules using silane coupling agents and attached the microcapsules to resin networks [80]. Subsequently, researchers evaluated the fatigue behavior of dental resin composites supplemented with 5 wt% and 25 wt% microcapsules. It was proven that the addition of 5 wt% siliconized microcapsules significantly improved fatigue crack extension resistance and that fatigue life was increased by 580 ± 15% [80].

In order to study the long-term fatigue behavior of self-healing composites, Ning et al. used two fatigue testing methods [100]. One was the traditional staircase test, after which the strength of all specimens was drastically reduced. However, the cyclic loading stresses in the test were much higher than the physiological loads in the oral cavity [101], so it was not possible to reveal potential improvements in fatigue resistance. On the contrary, the other was to use a custom-designed chewing simulator (Rub & Roll) to simulate the oral environment by imitating the cyclic chewing load in artificial saliva [102]. These findings proved that there was no decrease in the flexural strength of the SHDC, which confirmed the enhanced fatigue resistance of the SHDC. It also suggested that such a fatigue testing method involving cyclic loading under humid conditions was more in line with the actual clinical situation and was more conducive to the subsequent research and development of SHDCs.

## 4. Limitations and Challenges of Microcapsule-Based SHDCs

Nowadays, although dental self-healing polymers based on the existing microcapsule models have shown excellent advantages and have undergone significant advancements, there still remain several shortcomings and problems. Firstly, there is still no universally accepted standard for the size of microcapsules. Under-sized microcapsules will lead to insufficient self-healing agent content, making them unable to realize their full role. Meanwhile, a small contact area between capsules and resins can result in low mechanical interlocking effects, which can affect the effectiveness of self-healing. A study has confirmed that the incorporation of large-sized microcapsules into composite resins can effectively improve self-healing efficiency [63]. However, inadequate mechanical strength may be caused by long-term clinical applications. Therefore, determining the appropriate particle size range is a difficult problem that needs to be solved. Secondly, the presence of microcapsules in microcrack propagation is an important factor affecting self-healing performance, and it is critical to rupture and release the agents promptly. Exploring more suitable core and wall materials, and improving the adhesion between microcapsules and the matrix, such as by increasing the roughness of the shell and surface coupling treatments, are objectives that need to be achieved to enhance self-healing efficiency. Furthermore, the property of self-healing is closely linked to the addition of microcapsules. Applying insufficient amounts will lead to low self-healing efficiency. However, excessive microcapsules can affect the mechanical properties of the composites. Thus, it makes sense to find a delicate balance between mechanical properties and the self-healing efficiency, to minimize negative effects caused by the incorporation of microcapsules. In addition, although the synthesis process of microcapsules is mature and easy to apply in a polymer matrix, it is impossible to carry out secondary repair once microcapsules rupture. How to realize multiple repairs of microcapsule-based SHDCs, such as through the integration of different types of self-healing systems, will also become a research focus in the future. Furthermore, after the microcapsules have completed their functions, broken microcapsules in the composite resin may act as voids or defects, damaging the material’s mechanical strength over time. More attention should be paid to addressing this issue. A final point worth considering is the question of the biocompatibility of self-healing systems. Experiments have shown the good biocompatibility of PUF microcapsules and the non-cytotoxicity of composites containing microcapsules [62]. Nevertheless, there is potential for the release of unreacted free formaldehyde from PUF shells in the oral cavity [103]. Therefore, to deal with such a risk, it is necessary to conduct further in vivo and in vitro cytotoxicity testing.

## 5. Conclusions and Future Prospective

In summary, dental resin-based composites are complex systems, unlike structural and functional composites. They must maintain their chemical, structural, aesthetic and biological properties in the harsh oral environment. Although improvements have been made in the manufacturing and structural design of dental resin composites, the long-term success of this material is still limited by composite cracking and fracture. The microcapsule-based SHDC is recognized as a new material with potential and promise because it can self-sense damage and self-repair, simulating the self-curing phenomenon of organisms. Simultaneously, this novel SHDC can help the multifunctionality of the material be realized via the incorporation of remineralizing materials, antibacterial agents, and expanding monomers to achieve remineralization, antibacterial and anti-shrinkage functions. However, it is still in the conceptual research stage of preliminary in vitro trials, and more efforts are needed to transfer the concept of self-healing from the laboratory to dental restoration work. Therefore, to meet clinical needs, the continuous optimization and development of the self-healing systems, and an exploration of the balance between mechanical properties and self-healing efficiency while ensuring multifunctionality will be the focus of future research.

## Figures and Tables

**Table 1:** Summary of partial dental self-healing systems based on microcapsules.

Authors	Shell Material	Healing Liquid	Preparation Method	Composite Matrix Material	Self-Healing Efficiency	Significant Results
Wertzberger et al. (2010) [58]	PUF	DCPD + Grubbs’ catalyst	In situ polymerization	TEGDMA:UDMA:Bis-GMA (1:1:1) + silane 0.7 μ glass	57% (average)	The self-healing material was able to recover 57% of its original fracture toughness, but the modulus decreased.
Then et al. (2011) [59]	Melamine modified UF	DCPD	In situ polymerization	Bis-GMA:TEGDMA (7:3)	-	Melamine-modified UF microcapsules showed good adhesion to the dental host material. The small addition of microcapsules did not affect the performance of the matrix material.
Ouyang et al. (2011) [57]	PU	TEGDMA	Interfacial polycondensation	Commercial dental adhesive	-	These self-healing microcapsules not only exhibited a concentrated size distribution, high encapsulation efficiency, and good biocompatibility, but also improved the bond strength of the dental adhesive.
Wu et al. (2016) [62]	PUF	TEGDMA + DHEPT (BPO as initiator)	In situ polymerization	Bis-GMA:TEGDMA (1:1)	65% (15 wt%)	The self-healing resin containing 15 wt% microcapsules appeared to have good self-healing efficiency and cellular cytotoxicity without mechanical properties decreasing.
Huyang et al. (2016) [77]	Silanized silica	Aqueous solutions of polyacrylic acids	Silica condensation	Bis-GMA:HEMA (1:1) + strontium fluoroaluminasilicate glass powders	25% (average)	Salinization of the microcapsule surface can confer strong binding with methacrylic resins. SHDC containing 5 wt% microcapsules had the best overall performance.
Sharma et al. (2017) [81]	Silanized silica	Aqueous solutions of polyacrylic acids	Silica condensation	Bis-GMA:TEGDMA (1:1) + strontium fluoroaluminasilicate glass powders	-	Static mechanical results indicate that adding silane-modified microcapsules can improve hardness and flexural properties, but decreases the compressive strength of dental composite. Dynamic results indicate that the storage modulus decreased at 0–6 wt%, but increased at 9 wt%.
Yahyazadehfar et al. (2018) [80]	Silanized silica	Aqueous solutions of polyacrylic acids	Silica condensation	Bis-GMA:HEMA (1:1) + strontium fluoroaluminasilicate glass powders	24.2 ± 3.8% (5 wt%)	Two sets of SHDCs were prepared (separately with MA–silane and OH–silane). SHDCs containing 5 wt% MA–silane microcapsules achieve the best self-healing efficiency and mechanical properties, followed by increased fatigue crack growth resistance.
Ning et al. (2021) [63]	PUF	TEGDMA + DHEPT (BPO as initiator)	In situ polymerization	Commercially available flowable composite (Clearfil MajestyTM ES Flow)	76% (98 ± 43 μm)	The self-healing ability of flowable composite materials containing microcapsules increases with an increase in microcapsule size and concentration, as well as initiator concentration.
Althaqafi et al. (2022) [67]	PUF	TEGDMA + DHEPT (BPO as initiator)	In situ polymerization	Bis-GMA:TEGDMA (1:1) + SiO_2_ nanoparticles	-	Except that flexural strength decreased drastically with increasing microcapsule concentrations (>10 wt%) in the composites, other mechanical properties were not significantly affected.
Ahangaran et al. (2022) [76]	PMMA	TEGDMA + DHEPT (BPO as initiator)	Solvent evaporation	Bis-GMA:TEGDMA (7:3) + MPS-grafted SiO_2_ nanoparticles	78–121%	The incorporation of PMMA microcapsules into dental composites had excellent healing performance and antibacterial properties, without significant effects on flexural properties.
Fugolin et al. (2022) [72]	PUF	TEGDMA + DHEPT + DMAM (BPO as initiator)	In situ polymerization	Bis-GMA:Bis-EMA:UDMA:TEGDMA (2:2:2:1)	77.0–94.4%	The self-healing system incorporating DMAM provided obstacles to crack propagation, which translated into an increase in toughness and reversible bonding ability.
Moreira et al. (2022) [68]	PUF	TC_DHEPT_: TEGDMA + DHEPT BTC_DHEPT_: TEGDMA + Bis-GMA + DHEPT BTC_BPO_: TEGDMA + Bis-GMA + BPO	In situ polymerization	Bis-GMA:TEGDMA (1:1) + barium boroaluminosilicate glass particles	52.5% (TC_DHEPT_), 22.1% (BTC_BPO_ + BTC_DHEPT_)	The incorporation of microcapsules did not affect DC, flexural strength or elastic modulus. The self-healing efficiency was higher for the TC_DHEPT_ group than the BTC_BPO_ + BTC_DHEPT_ group.
Rozza et al. (2024) [82]	Silanized PUF	TEGDMA + DHEPT (BPO as initiator)	In situ polymerization	Bis-GMA:TEGDMA (1:1) + presilanized barium boroaluminosilicate glass particles	49–77%	

## Data Availability

No new data were created or analyzed in this study. Data sharing is not applicable to this article.
